# Disparities in time to treatment initiation for rectal cancer patients: an analysis of demographic and socioeconomic factors

**DOI:** 10.3389/fonc.2024.1327400

**Published:** 2024-05-10

**Authors:** Reed Popp, Shivam Bansal, Seema Sharan, Syeda Hoorulain Ahmed, Kulkaew Belle Sukniam, Swathi Raikot, Kyle Popp, Paola Berríos Jiménez, Harsheen Kaur Manaise, Gabrielle Kowkabany, Kristopher Attwood, Emmanuel M. Gabriel

**Affiliations:** ^1^ Department of Surgery, University of Florida College of Medicine, Gainesville, FL, United States; ^2^ Department of Surgery, Government Medical College and Hospital, Chandigarh, India; ^3^ Department of Surgery, Dow University of Health Sciences, Karachi, Pakistan; ^4^ Department of Surgery, Duke University Medical Center, Durham, NC, United States; ^5^ Department of Surgery, Mayo Clinic, Rochester, MN, United States; ^6^ Department of Surgery, Florida State University, Tallahassee, FL, United States; ^7^ Department of Surgery, University of Puerto Rico School of Medicine, San Juan, Puerto Rico; ^8^ Department of Surgery, The University of Alabama, Tuscaloosa, AL, United States; ^9^ Department of Surgery, Roswell Park Comprehensive Cancer Center, Buffalo, NY, United States; ^10^ Department of Surgery, Mayo Clinic, Jacksonville, FL, United States

**Keywords:** rectal cancer, treatment, disparity, socioeconomic factors, cancer care

## Abstract

**Background:**

This study investigated demographic and socioeconomic factors contributing to disparities in the time to treatment for rectal cancer. Subgroup analysis based on age < 50 and ≥ 50 was performed to identify differences in time to treatment among young adults (age < 50) compared to older adults with rectal cancer.

**Methods:**

An analysis was performed using data from the National Cancer Database, spanning from 2004 to 2019. The study encompassed 281,849 patients diagnosed with rectal cancer. We compared time intervals from diagnosis to surgery, radiation, and chemotherapy, considering age, sex, race, and socioeconomic variables. Analyses were performed for the entire cohort and for two subgroups based on age (< 50 and ≥ 50).

**Results:**

Overall, Hispanic patients experienced longer times to surgery, radiation, and chemotherapy compared to non-Hispanic patients (surgery: 94.2 *vs*. 79.1 days, radiation: 65.0 *vs*. 55.6 days, chemotherapy: 56.4 *vs*. 47.8 days, all p < 0.001). Patients with private insurance had shorter times to any treatment (32.5 days) compared to those with government insurance or no insurance (30.6 and 32.5 days, respectively, p < 0.001). Black patients experienced longer wait times for both radiation (63.4 days) and chemotherapy (55.2 days) compared to White patients (54.9 days for radiation and 47.3 days for chemotherapy, both p < 0.001). Interestingly, patients treated at academic facilities had longer times to treatment in surgery, radiation, and chemotherapy compared to those treated at comprehensive and community facilities. When analyzed by age, many of the overall differences persisted despite the age stratification, suggesting that these disparities were driven more by demographic and socioeconomic variables rather than by age.

**Conclusion:**

Significant differences in the time to treatment for rectal cancer have been identified. Hispanic patients, individuals lacking private insurance, Black patients, and patients receiving care at academic facilities had the longest times to treatment. However, these differences were largely unaffected by the age (< 50 and ≥ 50) subgroup analysis. Further investigation into the causes of these disparities is warranted to develop effective strategies for reducing treatment gaps and enhancing overall care for rectal cancer patients.

## Introduction

Colorectal cancer, ranking as the third most prevalent cancer among both men and women in the United States (excluding skin cancers), continues to be a major public health concern and is occurring at increasing frequency among young adults (1–6). In addressing the management of colorectal cancer, the National Comprehensive Cancer Network (NCCN) advocates for an individualized approach. For early-stage cases, the NCCN advocates a personalized approach that incorporates surgery, radiation therapy, and chemotherapy to address the unique needs of each patient, taking into account comorbidities, functionality, and frailty to determine how well the patient may tolerate any or all of these therapies. In locally advanced stages, the NCCN recommends a combination strategy, utilizing preoperative chemoradiotherapy followed by surgery to enhance the likelihood of a successful surgical resection with clear margins and minimized morbidity. In the context of advanced or metastatic stages, the NCCN emphasizes systemic chemotherapy as the primary treatment option. Additionally, considerations for targeted therapies and immune checkpoint inhibitors are advised in specific cases (5). In 2023, the American Cancer Society predicts that the United States will witness around 106,970 new cases of colon cancer and 46,050 new cases of rectal cancer. Colorectal cancer remains the third leading cause of cancer-related deaths among both genders in the United States. Over the past few decades, mortality rates associated with colorectal cancer have shown a consistent decline in both men and women (1–4). However, with the increasing incidence of rectal cancer among young adults, timely diagnosis and treatment are essential to achieving good outcomes for rectal cancer (7).

While there has been a decline in colorectal cancer mortality, disparities in cancer treatment and prognosis persist (5, 6, 8). Historically, overall survival among cancer patients has shown disparities across various racial and ethnic groups, with Black individuals experiencing the shortest overall survival compared to Asians and Whites (9). Black patients diagnosed with colorectal cancer at a younger age tend to receive delayed and suboptimal care compared to their White counterparts (10). Several factors, including patients’ insurance coverage, financial status, and demographic characteristics, contribute to longer time intervals to treatment (11). Furthermore, demographic factors and comorbidities explain only a small portion of this disparity, whereas the type of health insurance coverage accounts for a significant portion (28.6% for colon cancer and 19.4% for rectal cancer). This suggests that enhancing access to healthcare could potentially help reduce the disparities in cancer outcomes between racial groups. Examining the socioeconomic and demographic factors linked to longer times in initiating rectal cancer treatment, this study aimed to identify disparities related to the time (in days) to comprehensive cancer care (including surgery, radiation, and chemotherapy), with particular emphasis on age-based disparities (age < 50 and ≥ 50). To our knowledge, this study represents the largest cohort examining differences in time to treatment for rectal cancer, particularly with respect to assessing disparities among younger patients (age < 50). Shorter time to treatment of rectal cancer has been shown by prior to studies to be associated with improved survival outcomes (12, 13). Recognizing the importance of addressing these disparities, efforts should be directed towards narrowing the accessibility gap and ensuring timely access to appropriate medical care for rectal cancer patients.

## Patients and methods

We conducted a retrospective study using the National Cancer Database (NCDB) between 2004 and 2019. Because the NCDB is a nationally available, deidentified dataset, Institutional Review Board approval was not required for our study, which focused on individuals aged 18 and older who were eligible for inclusion. Patients with rectal cancer, as defined as cancer located within 12 cm of the anal verge by rigid proctoscopy (5), coded by the following ICD-O-3 codes (8140–8148, 8200, 8260–8263, and 8480–8496), and staged according to the American Joint Committee on Cancer (AJCC 6th and 7th edition) guidelines, were included. Participants with missing information were excluded from the analysis.

Variables in the analysis included age (< 40, 40-50, 50-60, 60-70, > 70), sex (male, female), race (White, Black, Native American, Asian, other), Hispanic origin, insurance status (uninsured, private, government), income (< $63,000 and > $63,000, as predetermined by the NCDB based on neighborhood or zip code analysis), treatment facility type (community, comprehensive, academic, other also using predefined definitions from the NCDB), and geographic location (rural, metropolitan, urban). Times to actual treatment (surgery, chemotherapy, and/or radiation) were computed and summarized. The NCDB records whether a patient received these treatments, but does not indicate eligibility for treatment in the cases where no treatment (i.e., surgery, chemotherapy, and/or radiation) was received. The time to a specific treatment (in days) was defined as when that treatment was first received (e.g., receipt of chemotherapy as neoadjuvant, adjuvant, or peri-operative).

Overall, the NCDB is thought to capture approximately 70% of the cancer patients treated within the US for several cancer malignancies (5, 14). Each site specific dataset contains over 200 variables, ranging widely from demographic, socioeconomic, pathologic, and treatment related variables, including times to treatment for first initial therapy as well as second and third line therapies utilized commonly in the multidisciplinary approach to rectal cancer. The NCDB has been utilized extensively by many investigators, including our group, to analyze disparities in cancer care across many different cancers (15–19).

Statistical analysis was performed using SAS version 9.4 (SAS Institute Inc., Cary, NC, USA). The clinical and demographic characteristics among each treatment variable (time to any treatment, time to surgery, time to radiation, and time to chemotherapy) were summarized. The means and standard deviations were provided for continuous variables and analyzed using ANOVA. Analyses were performed for the entire cohort and for the two subgroups based on age (age < 50 and ≥ 50). The threshold for statistical significance was set at a p-value of 0.05.

## Results

### Time to first treatment

The study sample for time to first treatment comprised 281,849 patients. Younger patients (under 40) experienced the shortest time, averaging 25.3 days (**Table 1**). Wait times increased with age. White patients had the shortest average waiting period (28.8 days), followed by Asians (30.0 days), Black patients (31.5 days), and Native Americans (32.9 days). Hispanic patients generally waited longer (35.6 days) than non-Hispanic patients (28.8 days). Academic institutions had the longest average wait times (33.5 days), while community facilities had a shorter average wait time of 26.6 days. Patients with private insurance waited an average of 26.5 days. When analyzed by age, many of these disparities persisted regardless of the age subgroup (< 50 and ≥ 50), as shown in **Table 2**. Differences in time to first treatment among patients in urban or rural locations did not reach statistical significance among the age < 50 group (p = 0.08).

**Table 1:** Time (in days) to first treatment (including surgery, chemotherapy, and/or radiation).

		n	Mean (SD)	P-value
Age (years)	< 40	11012	25.3 (30.2)	< 0.001
	40-50	32744	27.3 (30.8)	
	50-60	73529	27.7 (35.1)	
	60-70	74160	30.0 (33.4)	
	> 70	90404	30.1 (34.8)	
Sex	Male	164914	29.8 (34.8)	< 0.001
	Female	116935	27.7 (32.7)	
Race	White	237077	28.5 (32.6)	< 0.001
	Black	28753	31.5 (40.3)	
	Native American	1204	32.9 (33.2)	
	Asian	8019	30.0 (39.2)	
	Other	4065	31.4 (41.2)	
Hispanic Origin	Yes	4728	35.6 (39.3)	< 0.001
	No	250871	28.8 (33.6)	
Rural/Urban	Metro	225803	28.7 (34.6)	< 0.001
	Urban	41626	29.6 (31.0)	
	Rural	5807	28.7 (28.5)	
Insurance Status	Not Insured	10702	32.5 (47.2)	< 0.001
	Private	124879	26.5 (30.7)	
	Government	140767	30.6 (35.1)	
	Unknown	5501	33.3 (38.2)	
Income	< $63,000	170743	29.4 (34.7)	< 0.001
	> $63,000	86817	27.5 (32.5)	
Grade	Well	29610	24.3 (35.4)	< 0.001
	Moderately	156751	31.2 (32.2)	
	Poorly	30167	29.9 (32.3)	
	Undifferentiated	3291	26.5 (27.5)	
Stage	0	12807	11.3 (30.5)	< 0.001
	I	48276	25.5 (35.9)	
	II	47887	37.0 (31.2)	
	III	54361	36.8 (28.6)	
	IV	34126	32.5 (33.3)	
Facility Type	Community	26622	26.6 (31.9)	< 0.001
	Comprehensive	114782	26.6 (31.2)	
	Academic	90799	33.5 (36.9)	
	Other	38634	27.6 (35.7)	

A total of 281,849 patients was eligible for analysis.

**Table 2:** Time (in days) to first treatment (including surgery, chemotherapy, and/or radiation), analyzed by age < 50 years and age ≥ 50 years.

Age < 50 years		n	Mean (SD)	P-value
Sex	Male	24367	27.58 (31.04)	< 0.001
	Female	19389	25.72 (30.14)	
Race	White	35219	26.20 (28.30)	< 0.001
	Black	5297	29.27 (38.79)	
	Native American	260	30.28 (30.98)	
	Asian	1500	27.61 (30.95)	
	Other	952	30.88 (44.14)	
Hispanic Origin	Yes	1082	31.86 (33.81)	< 0.001
	No	37855	26.49 (30.11)	
Rural/Urban	Metro	35917	26.55 (30.74)	0.08
	Urban	5685	27.52 (29.45)	
	Rural	664	26.66 (25.04)	
Insurance Status	Not Insured	3002	30.08 (33.75)	< 0.001
	Private	31814	25.22 (28.61)	
	Government	7852	30.96 (35.71)	
	Unknown	1088	32.17 (35.56)	
Income	< $63,000	25150	27.39 (31.67)	< 0.001
	> $63,000	14510	25.19 (28.32)	
Grade	Well	4609	22.80 (35.75)	< 0.001
	Moderately	23763	28.82 (28.65)	
	Poorly	5207	27.94 (27.75)	
	Undifferentiated	547	23.84 (21.90)	
Stage	0	1354	9.97 (28.56)	< 0.001
	I	6186	22.31 (33.60)	
	II	6477	32.20 (25.74)	
	III	11235	33.61 (27.69)	
	IV	6782	29.22 (28.88)	
Facility Type	Community	2701	25.73 (34.86)	< 0.001
	Comprehensive	12580	24.20 (26.66)	
	Academic	12931	31.25 (32.81)	
	Other	4532	25.27 (31.83)	
Age ≥ 50 years		n	Mean (SD)	P-value
Sex	Male	140547	30.12 (35.34)	< 0.001
	Female	97546	28.09 (33.20)	
Race	White	201858	28.89 (33.33)	< 0.001
	Black	23456	32.04 (40.64)	
	Native American	944	33.58 (33.78)	
	Asian	6519	30.59 (40.88)	
	Other	3113	31.51 (40.23)	
Hispanic Origin	Yes	3646	36.67 (40.68)	< 0.001
	No	213016	29.17 (34.13)	
Rural/Urban	Metro	189886	29.12 (35.25)	< 0.001
	Urban	35941	29.96 (31.20)	
	Rural	5143	28.99 (28.89)	
Insurance Status	Not Insured	7700	33.47 (51.42)	< 0.001
	Private	93065	26.89 (31.35)	
	Government	132915	30.58 (35.11)	
	Unknown	4413	33.63 (38.77)	
Income	< $63,000	145593	29.76 (35.19)	< 0.001
	> $63,000	72307	27.94 (33.24)	
Grade	Well	25001	24.57 (35.34)	< 0.001
	Moderately	132988	31.67 (32.79)	
	Poorly	24960	30.32 (33.14)	
	Undifferentiated	2744	26.97 (28.42)	
Stage	0	11453	11.41 (30.70)	< 0.001
	I	42090	25.93 (36.18)	
	II	41410	37.74 (31.94)	
	III	43126	37.62 (28.71)	
	IV	27344	33.34 (34.30)	
Facility Type	Community	23921	26.66 (31.56)	< 0.001
	Comprehensive	102202	26.92 (31.75)	
	Academic	77868	33.81 (37.50)	
	Other	34102	27.89 (36.14)	

A total of 281,849 patients was also eligible for analysis.

### Time to surgery

The study sample for time to surgery included 233,332 patients. Males waited longer for definitive surgery (83.2 days), while females waited an average of 73.1 days (**Table 3**). Patients of Native American origin had the longest waiting period (96.5 days), followed by White patients (79.4 days), Asians (79.0 days), and Black patients (74.9 days). Urban residents had longer intervals (83.7 days) compared to metropolitan (77.8 days) and rural residents (81.9 days). Uninsured patients faced the longest waiting time (99.1 days), while those with private insurance waited 81.1 days. Academic facilities had the lengthiest waiting period (90.5 days), compared to community facilities (67.6 days) and comprehensive facilities (72.1 days). With regard to age-based disparities (**Table 4**), no new differences were identified from the overall cohort, and disparities within each variable all achieved statistical significance.

**Table 3:** Time (in days) to surgery.

		n	Mean (SD)	P-value
Age (years)	< 40	9235	89.4 (81.3)	< 0.001
	40-50	27496	91.4 (79.6)	
	50-60	62724	82.2 (79.6)	
	60-70	62126	83.1 (78.0)	
	> 70	71751	66.6 (73.1)	
Sex	Male	136155	83.2 (78.8)	< 0.001
	Female	97177	73.1 (76.1)	
Race	White	196980	79.4 (76.5)	< 0.001
	Black	22759	74.9 (85.6)	
	Native American	984	96.5 (83.0)	
	Asian	6951	79.0 (80.7)	
	Other	3379	86.9 (85.5)	
Hispanic Origin	Yes	3787	94.2 (87.3)	< 0.001
	No	207758	79.1 (77.5)	
Rural/Urban	Metro	186902	77.8 (78.3)	< 0.001
	Urban	34419	83.7 (75.1)	
	Rural	4763	81.9 (72.7)	
Insurance Status	Not Insured	7735	99.1 (87.9)	< 0.001
	Private	108952	81.1 (76.4)	
	Government	112574	75.8 (78.1)	
	Unknown	4071	74.5 (77.2)	
Income	< $63,000	139531	78.8 (77.5)	< 0.001
	> $63,000	73012	76.4 (77.0)	
Grade	Well	26784	58.0 (71.9)	< 0.001
	Moderately	133928	87.4 (76.4)	
	Poorly	23125	82.9 (75.6)	
	Undifferentiated	2752	70.0 (71.0)	
Stage	0	12582	18.5 (41.8)	< 0.001
	I	45102	47.0 (58.2)	
	II	39629	121.2 (64.5)	
	III	45259	132.1 (63.9)	
	IV	12029	132.1 (116.3)	
Facility Type	Community	20796	67.60 (74.0)	< 0.001
	Comprehensive	95509	72.1 (72.8)	
	Academic	75030	90.5 (83.6)	
	Other	32762	77.1 (75.8)	

A total of 233,332 patients was eligible for analysis.

**Table 4:** Time (in days) to surgery, analyzed by age < 50 years and age ≥ 50 years.

Age < 50 years		n	Mean (SD)	P-value
Sex	Male	20171	97.08 (81.26)	< 0.001
	Female	16560	83.39 (77.81)	
Race	White	29733	91.99 (78.34)	< 0.001
	Black	4251	82.89 (87.91)	
	Native American	211	110.09 (91.98)	
	Asian	1320	91.10 (77.60)	
	Other	781	95.99 (84.38)	
Hispanic Origin	Yes	858	103.49 (89.66)	< 0.001
	No	31924	90.85 (79.36)	
Rural/Urban	Metro	30107	89.74 (80.09)	< 0.001
	Urban	4772	96.03 (79.18)	
	Rural	564	93.47 (74.16)	
Insurance Status	Not Insured	2200	100.13 (84.10)	< 0.001
	Private	27731	88.64 (77.18)	
	Government	6014	99.30 (89.87)	
	Unknown	786	80.72 (79.57)	
Income	< $63,000	20728	91.05 (80.68)	0.002
	> $63,000	12444	88.29 (77.93)	
Grade	Well	4249	63.56 (75.62)	< 0.001
	Moderately	20546	101.73 (76.76)	
	Poorly	3931	99.97 (79.32)	
	Undifferentiated	459	87.57 (80.53)	
Stage	0	1336	19.51 (44.69)	< 0.001
	I	5891	47.60 (59.48)	
	II	5758	122.42 (57.06)	
	III	9864	134.29 (60.35)	
	IV	2779	150.70 (114.14)	
Facility Type	Community	2145	80.36 (77.35)	< 0.001
	Comprehensive	10650	82.72 (72.92)	
	Academic	10787	102.89 (85.45)	
	Other	3914	89.56 (77.22)	
Age ≥ 50 years		n	Mean (SD)	P-value
Sex	Male	115984	80.78 (78.05)	< 0.001
	Female	80617	71.02 (75.51)	
Race	White	167247	77.17 (75.96)	< 0.001
	Black	18508	73.05 (84.96)	
	Native American	773	92.73 (80.07)	
	Asian	5631	76.10 (81.16)	
	Other	2598	84.13 (85.65)	
Hispanic Origin	Yes	2929	91.50 (86.36)	< 0.001
	No	175834	76.91 (76.99)	
Rural/Urban	Metro	156795	75.47 (77.78)	< 0.001
	Urban	29647	81.75 (74.18)	
	Rural	4199	80.39 (72.34)	
Insurance Status	Not Insured	5535	98.73 (89.34)	< 0.001
	Private	81221	78.46 (76.01)	
	Government	106560	74.47 (77.17)	
	Unknown	3285	73.02 (76.57)	
Income	< $63,000	118803	76.67 (76.75)	< 0.001
	> $63,000	60568	73.97 (76.57)	
Grade	Well	22535	56.96 (71.10)	< 0.001
	Moderately	113382	84.85 (76.10)	
	Poorly	19194	79.45 (74.38)	
	Undifferentiated	2293	66.49 (68.38)	
Stage	0	11246	18.38 (41.41)	< 0.001
	I	39211	46.89 (58.04)	
	II	33871	121.04 (65.66)	
	III	35395	131.46 (64.84)	
	IV	9250	126.40 (116.34)	
Facility Type	Community	18651	66.18 (73.45)	< 0.001
	Comprehensive	84859	70.71 (72.63)	
	Academic	64243	88.47 (83.15)	
	Other	28848	75.42 (75.49)	

A total of 233,332 patients was also eligible for analysis.

### Time to radiation

The study sample for time to radiation included 118,969 patients. Black patients had the longest waiting period (63.4 days), followed by Asians (59.7 days), White patients (54.9 days), and Native Americans (50.2 days) (**Table 5**). Hispanic patients generally waited longer (65.0 days) than non-Hispanic patients (55.6 days). Patients with government insurance waited longer (57.4 days) than uninsured patients (56.3 days). Income levels did not significantly affect the time to receive radiation treatment. These findings were replicated with the subgroup age analysis as listed in **Table 6**.

**Table 5:** Time (in days) to radiation.

		n	Mean (SD)	P-value
Age (years)	< 40	5391	53.5 (56.8)	< 0.001
	40-50	17022	53.5 (56.8)	
	50-60	33351	55.4 (55.8)	
	60-70	33627	57.4 (58.9)	
	> 70	29578	56.4 (53.3)	
Sex	Male	72899	55.3 (56.9)	< 0.001
	Female	46070	56.8 (55.4)	
Race	White	102823	54.9 (55.3)	< 0.001
	Black	9788	63.4 (65.2)	
	Native American	562	50.2 (45.3)	
	Asian	3283	59.7 (55.4)	
	Other	1687	59.1 (58.1)	
Hispanic Origin	Yes	2104	65.0 (60.7)	< 0.001
	No	106208	55.6 (56.3)	
Rural/Urban	Metro	92781	56.6 (57.9)	< 0.001
	Urban	19614	52.8 (49.1)	
	Rural	2747	51.1 (48.3)	
Insurance Status	Not Insured	5036	56.3 (55.6)	< 0.001
	Private	58494	54.4 (56.1)	
	Government	53307	57.4 (56.5)	
	Unknown	2132	55.5 (59.3)	
Income	< $63,000	72272	55.4 (56.0)	0.032
	> $63,000	35337	56.2 (56.4)	
Grade	Well	8855	55.9 (53.2)	< 0.001
	Moderately	77759	56.3 (56.0)	
	Poorly	14048	59.0 (59.5)	
	Undifferentiated	1393	63.1 (61.0)	
Stage	0	505	79.3 (80.1)	< 0.001
	I	11178	70.0 (68.4)	
	II	33243	45.3 (41.1)	
	III	40826	47.4 (40.8)	
	IV	7137	89.6 (97.8)	
Facility Type	Community	10367	55.4 (54.9)	< 0.001
	Comprehensive	48381	53.5 (54.7)	
	Academic	38362	59.4 (58.1)	
	Other	16468	55.4 (56.9)	

A total of 118,969 patients was eligible for analysis.

**Table 6:** Time (in days) to radiation, analyzed by age < 50 years and age ≥ 50 years.

Age < 50 years		n	Mean (SD)	P-value
Sex	Male	13096	52.35 (56.02)	< 0.001
	Female	9317	55.12 (57.87)	
Race	White	18727	52.54 (55.84)	< 0.001
	Black	2149	59.23 (62.06)	
	Native American	138	52.85 (47.87)	
	Asian	771	55.73 (54.99)	
	Other	447	56.28 (58.59)	
Hispanic Origin	Yes	556	61.50 (65.01)	< 0.001
	No	19526	53.30 (56.96)	
Rural/Urban	Metro	18089	53.96 (57.52)	0.001
	Urban	3158	51.39 (53.35)	
	Rural	374	45.20 (46.35)	
Insurance Status	Not Insured	1543	54.74 (57.28)	< 0.001
	Private	16671	52.43 (55.40)	
	Government	3704	57.48 (60.84)	
	Unknown	495	55.82 (68.34)	
Income	< $63,000	12903	53.22 (56.77)	0.82
	> $63,000	7260	53.41 (56.73)	
Grade	Well	1586	53.14 (57.70)	0.002
	Moderately	14488	53.51 (54.51)	
	Poorly	2880	56.36 (64.03)	
	Undifferentiated	303	63.62 (69.36)	
Stage	0	69	71.75 (71.85)	< 0.001
	I	1633	66.19 (65.49)	
	II	5188	41.18 (36.21)	
	III	9198	45.07 (41.18)	
	IV	1833	95.74 (98.02)	
Facility Type	Community	1322	52.26 (55.16)	< 0.001
	Comprehensive	6652	49.74 (52.87)	
	Academic	6591	58.37 (62.50)	
	Other	2457	51.28 (50.79)	
Age ≥ 50 years		n	Mean (SD)	P-value
Sex	Male	59803	55.90 (57.03)	< 0.001
	Female	36753	57.18 (54.74)	
Race	White	84096	55.47 (55.16)	< 0.001
	Black	7639	64.61 (65.99)	
	Native American	424	49.38 (44.40)	
	Asian	2512	60.92 (55.52)	
	Other	1240	60.05 (57.95)	
Hispanic Origin	Yes	1548	66.28 (58.99)	< 0.001
	No	86682	56.11 (56.17)	
Rural/Urban	Metro	74692	57.21 (58.02)	< 0.001
	Urban	16456	53.10 (48.23)	
	Rural	2373	52.07 (48.56)	
Insurance Status	Not Insured	3493	56.92 (54.78)	< 0.001
	Private	41823	55.16 (56.32)	
	Government	49603	57.41 (56.11)	
	Unknown	1637	55.40 (56.33)	
Income	< $63,000	59369	55.91 (55.84)	0.011
	> $63,000	28077	56.94 (56.28)	
Grade	Well	7269	56.44 (52.11)	< 0.001
	Moderately	63271	56.89 (56.34)	
	Poorly	11168	59.66 (58.27)	
	Undifferentiated	1090	62.94 (58.45)	
Stage	0	436	80.51 (81.34)	< 0.001
	I	9545	70.66 (68.85)	
	II	28055	46.10 (41.89)	
	III	31628	48.12 (40.61)	
	IV	5304	87.52 (97.63)	
Facility Type	Community	9045	55.80 (54.87)	< 0.001
	Comprehensive	41729	54.14 (55.00)	
	Academic	31771	59.62 (57.12)	
	Other	14011	56.11 (57.91)	

A total of 118,969 patients was also eligible for analysis.

### Time to chemotherapy

Lastly, the study sample for time to chemotherapy included 169,618 patients. Black patients had the longest time to chemotherapy treatment (55.2 days), while Asians, Whites, and Native Americans had shorter average waiting periods (49.7 days, 47.3 days, and 46.8 days, respectively) (**Table 7**). Patients of Hispanic origin experienced longer times (56.4 days) compared to their non-Hispanic counterparts (47.8 days). When analyzed by age, these racial and ethnic differences persisted regardless of the age subgroup (**Table 8**).

**Table 7:** Time (in days) to chemotherapy.

		n	Mean (SD)	P-value
Age (in years)	< 40	7646	41.6 (40.0)	< 0.001
	40-50	23413	43.9 (49.4)	
	50-60	46145	47.5 (47.9)	
	60-70	47325	50.0 (52.1)	
	> 70	45089	50.2 (46.8)	
Sex	Male	103634	47.9 (47.4)	0.005
	Female	65984	48.6 (50.8)	
Race	White	144939	47.3 (48.4)	< 0.001
	Black	15673	55.2 (54.3)	
	Native American	794	46.8 (37.0)	
	Asian	4459	49.7 (41.9)	
	Other	2441	49.5 (46.0)	
Hispanic Origin	Yes	3109	56.4 (53.7)	< 0.001
	No	151242	47.8 (47.4)	
Rural/Urban	Metro	133360	48.5 (50.4)	< 0.001
	Urban	27063	47.0 (42.8)	
	Rural	3819	44.8 (37.2)	
Insurance Status	Not Insured	8074	49.3 (55.3)	< 0.001
	Private	78323	45.5 (45.5)	
	Government	79693	50.5 (51.1)	
	Unknown	3528	49.5 (48.2)	
Income	< $63,000	104051	48.6 (51.1)	< 0.001
	> $63,000	50100	47.0 (45.2)	
Grade	Well	11753	49.4 (51.2)	< 0.001
	Moderately	103494	49.2 (48.5)	
	Poorly	21692	47.8 (43.5)	
	Undifferentiated	2115	50.5 (43.1)	
Stage	0	709	66.7 (91.8)	< 0.001
	I	14182	62.7 (70.1)	
	II	40688	44.9 (39.2)	
	III	49780	44.1 (36.6)	
	IV	30127	41.4 (40.0)	
Facility Type	Community	15756	48.2 (48.6)	< 0.001
	Comprehensive	68044	46.4 (45.2)	
	Academic	55549	51.2 (47.6)	
	Other	22623	47.9 (62.5)	

A total of 169,618 patients was eligible for analysis.

**Table 8:** Time (in days) to chemotherapy, analyzed by age < 50 years and age ≥ 50 years.

Age < 50 years		n	Mean (SD)	P-value
Sex	Male	18129	42.41 (41.43)	< 0.001
	Female	12930	44.62 (54.40)	
Race	White	25594	42.35 (47.34)	< 0.001
	Black	3299	50.47 (49.21)	
	Native American	196	45.72 (39.11)	
	Asian	1008	41.53 (32.69)	
	Other	659	45.48 (44.65)	
Hispanic Origin	Yes	808	51.63 (52.90)	< 0.001
	No	26901	42.76 (46.81)	
Rural/Urban	Metro	25194	43.65 (48.84)	0.023
	Urban	4273	42.62 (40.58)	
	Rural	508	38.37 (30.75)	
Insurance Status	Not Insured	2397	46.55 (43.92)	< 0.001
	Private	22124	41.84 (48.50)	
	Government	5746	47.52 (43.53)	
	Unknown	792	44.96 (46.25)	
Income	< $63,000	18135	44.36 (51.47)	< 0.001
	> $63,000	9959	41.21 (41.14)	
Grade	Well	2058	43.81 (41.97)	0.24
	Moderately	18948	44.34 (50.28)	
	Poorly	4394	42.78 (43.35)	
	Undifferentiated	440	42.42 (34.17)	
Stage	0	106	62.90 (56.57)	< 0.001
	I	2079	60.43 (65.16)	
	II	5990	40.54 (35.94)	
	III	10701	39.98 (34.85)	
	IV	6343	37.04 (35.28)	
Facility Type	Community	1888	44.25 (53.38)	< 0.001
	Comprehensive	8973	41.19 (39.53)	
	Academic	9322	46.74 (44.14)	
	Other	3230	42.94 (77.66)	
Age ≥ 50 years		n	Mean (SD)	P-value
Sex	Male	85505	49.03 (48.54)	0.08
	Female	53054	49.51 (49.87)	
Race	White	119345	48.36 (48.55)	< 0.001
	Black	12374	56.41 (55.56)	
	Native American	598	47.15 (36.25)	
	Asian	3451	52.03 (43.95)	
	Other	1782	51.03 (46.36)	
Hispanic Origin	Yes	2301	58.03 (53.82)	< 0.001
	No	124341	48.90 (47.40)	
Rural/Urban	Metro	108166	49.67 (50.70)	< 0.001
	Urban	22790	47.78 (43.09)	
	Rural	3311	45.80 (38.00)	
Insurance Status	Not Insured	5677	50.39 (59.44)	< 0.001
	Private	56199	46.97 (44.16)	
	Government	73947	50.77 (51.59)	
	Unknown	2736	50.81 (48.73)	
Income	< $63,000	85916	49.52 (50.93)	< 0.001
	> $63,000	40141	48.46 (46.02)	
Grade	Well	9695	50.57 (52.91)	0.002
	Moderately	84546	50.25 (48.08)	
	Poorly	17298	49.08 (43.49)	
	Undifferentiated	1675	52.63 (44.89)	
Stage	0	603	67.31 (96.66)	< 0.001
	I	12103	63.13 (70.89)	
	II	34698	45.65 (39.67)	
	III	39079	45.18 (36.92)	
	IV	23784	42.52 (41.05)	
Facility Type	Community	13868	48.79 (47.86)	< 0.001
	Comprehensive	59071	47.19 (45.98)	
	Academic	46227	52.11 (48.20)	
	Other	19393	48.76 (59.53)	

A total of 169,618 patients was also eligible for analysis.

## Discussion

Through our analysis, we have identified several socioeconomic and demographic factors that were associated with longer time intervals between the diagnosis and treatment of rectal cancer. The time to first treatment for rectal cancer may be an important interval when determining how the time interval of treatment may impact patient outcomes. Some studies have reported that longer diagnostic time intervals might not necessarily be associated with a worse prognosis (20). One meta-analysis found that, in their analysis of 40 studies involving over 20,000 patients, most studies showed no link between time to the treatment of colorectal cancer and survival rates (21). While survival rates may not be affected, however, early diagnosis and treatment remain critical as longer times to treatment can have psychological ramifications for patients. The uncertainty and anxiety linked to a postponed diagnosis can cause distress for both patients and their families. Survivors who perceive a diagnostic delay have been found to experience higher levels of cancer-related distress (22). They may also face challenges in terms of social support, as the prolonged duration of the treatment process can strain relationships and create a sense of isolation. These hurdles can have a profound effect on the patient’s mental health and their ability to cope with the demands associated with cancer diagnosis and treatment.

Notably, the ethnicity of the patients was associated with the timing of when therapeutic interventions like radiation, chemotherapy, and surgery were initiated. We found that Hispanic patients had longer time intervals before receiving these treatments compared to non-Hispanic patients, highlighting a potential disparity in treatment timing across various treatment modalities. Among young patients < 50, this finding persisted with approximately the same length of time compared to those ≥ 50. Overall this may raise concern, especially in the context of the substantial rise in colorectal cancer incidence among Hispanics aged 50-59, while the incidence in other racial and ethnic groups has remained stable (20). Language barriers, which can impede effective communication between healthcare providers and Hispanic patients, may be a key element exacerbating this disparity (23). The quality of physician-patient communication has been closely linked to language, with one study showing that Spanish-speaking patients expressed lower levels of satisfaction with the communication they received (23). Factors such as diverse cultural beliefs, immigration status, and limited access to healthcare due to lack of insurance further may hinder Hispanic patients’ ability to seek and receive proper healthcare (24).

The time to initiate treatment for rectal cancer also was associated with the patient’s racial background. Across all treatment modalities, except for surgery, Black patients experienced significantly longer intervals before receiving their first treatment compared to White patients, which was found regardless of the age subgroup analysis. Our results align with Robbins et al.’s findings, which showed that even after controlling for colorectal cancer screening and diagnosis rates, longer time intervals in receiving adjuvant therapy could still be observed among Black patients (21). This may imply that factors beyond screening and diagnosis could be associated with longer times to administration of necessary adjuvant therapies in Black patients. This could be attributed in part to a potential disparity in specialist consultations between Black and White patients. Black patients have been shown to have lower rates of consultation with medical oncologists and surgical oncologists compared to Whites (25). Disparities in cancer consultations carry consequences, particularly with respect to timely access to essential treatments such as surgery and chemotherapy. The absence of adequate referrals to specialists may lead to insufficient consideration of multimodality therapy (26). Many cancer cases require a combination of treatments, including surgery, chemotherapy, and radiation therapy. When Black patients experience lower rates of consultation, the opportunity to discuss and implement comprehensive multimodality therapy plans may be missed. This can affect the overall quality of care and the chances of achieving the best possible treatment outcomes. Moreover, it is important to note that the underlying mechanisms responsible for this disparity are complex and warrant further investigation. Efforts should be focused on understanding and addressing the barriers that contribute to the delayed initiation of treatment among Black patients. This may involve interventions to improve access to healthcare services, increase awareness and education about the importance of early treatment, and promote culturally sensitive care to ensure equitable and timely delivery of adjuvant therapies.

We also identified an association between the patient’s income level and the time intervals in rectal cancer treatment. Across all treatment modalities, except for radiation, individuals with an annual income below $63,000 experienced longer time intervals before receiving their initial rectal cancer treatment compared to their counterparts with incomes surpassing this threshold. The proportions of patients making < $63,000 and > $63,000 were very similar between the age < 50 and ≥ 50 subgroups, and both age groups < $63,000 experienced similar time intervals. This observation may be influenced by various factors, such as affordability challenges in paying health insurance premiums, difficulties in applying for Medicaid, postponing physician visits due to high co-pays, declining diagnostic testing due to cost, and concerns about work interference, all of which may be disproportionately more burdensome on younger patients (26). While our study does not provide causal association between income and time to treatment, these findings may underscore a need for interventions aimed at rectifying potential imbalances and ensuring equitable healthcare outcomes for all patients, regardless of their socioeconomic status.

It is vital that healthcare providers and policymakers collaborate to institute reforms that address the socioeconomic barriers faced by patients with lower incomes. This may include implementing policies that expand access to affordable healthcare coverage, subsidizing treatment costs for economically disadvantaged individuals, and fostering partnerships with community organizations to bridge the financial gap. By proactively addressing the link between income and longer times to treatment, we can work towards a healthcare landscape that prioritizes accessibility and equity, ensuring that individuals of all income levels have equitable opportunities to receive timely and life-saving rectal cancer treatment.

Beyond factors like race/ethnicity and socioeconomic status, the type of healthcare facility where patients receive treatment may also contribute to treatment disparities. When considering all types of treatment, individuals undergoing rectal cancer treatment at academic institutions had longer time intervals before their initial treatment compared to those treated at comprehensive and community facilities. Similar findings were yielded by Schmerhorn et al. in their study conducted on breast cancer patients. The research revealed that longer time intervals to treatment in breast cancer treatment were primarily linked to the decision to receive care at academic institutions (11). Factors such as educational level, comorbidity burden, and insurance status accounted for 11%, 8%, and 13% of the variation in treatment timing among Black, Hispanic, and other non-White patients, respectively (11).

Additionally, the timing of a patient’s initial rectal cancer treatment was associated with their insurance status. In every treatment type, our results showed that individuals with private insurance experienced shorter waiting times for treatment compared to those without insurance or relying on government insurance. This aspect of our research highlights the influence of diverse insurance coverage on healthcare results, revealing that patients with private insurance experience expedited access to treatments for rectal cancer, while individuals lacking insurance or reliant on government insurance encounter prolonged time intervals. Younger patients would not be expected to have of Medicare, which is most often based on age > 65 years old. Indeed, in our analysis, most patients under 50 had private insurance. But there were still a few thousand patients < 50 who were uninsured, and those patients also experienced longer wait times similar to the cohort as a whole.

Within the differences we have identified in the mean times to treatment for rectal cancer, it is also interesting to note that similar differences were identified in the variances (standard deviations) of many comparisons. For example, minority patients, including Black and Hispanic patients, had wider treatment time distributions than White and non-Hispanic patients. It may be the case that underrepresented patients are more likely to experience greater variability in their time to treatment. In addition, treatment at academic facilities overall had the high variance in time to treatment. However, it may have been the case that patients who received care at academic centers tended to have more advanced disease and required additional workup, which would have prolonged the timing of initial treatment. Thus, it important to note that limitations in the NCDB and our analysis preclude forming any causal associations among the differences that we observed.

Indeed, it is crucial to bear in mind that the data available in the NCDB has specific limitations, necessitating further in-depth analysis. Our study, being retrospective and based on a comprehensive examination of a large database, was subject several constraints, including error in data input. The reliability and generalizability of our findings may also be compromised by missing data, which was excluded. In addition, while many of the results were found to be statistically significant, likely because of the large cohort of patients, not all of the differences in time intervals would be expected to be clinically significant. We have highlighted the largest differences in time intervals, but many other differences were identified that were under 10 days. These differences would be less likely to be associated with suboptimal care than potentially longer time to treatment differences. The NCDB is also limited in distinguishing time intervals between multimodal treatment. Times to treatment overall represent time to the first treatment, but are not organized based on the steps in multidisciplinary care.

In conclusion, our study brings attention to differences in the time to rectal cancer treatment among different demographic and socioeconomic variables. While exploratory, the results of our study provides some insight into a large number of factors that are associated with the timely initiation of treatment for rectal cancer. When assessed by age, younger patients with rectal cancer overall were found to have similar differences to the older subgroup, suggesting that these disparities were driven more by demographic and socioeconomic variables rather than by age. Additional obstacles, such as affordability challenges in paying health insurance premiums, applying for Medicaid, high co-pays, and concerns about work interference, may also exist and were not specifically captured by our analysis. Nonetheless, healthcare and policy professionals should prioritize awareness of these factors and actively support rectal cancer patients, ensuring they receive adequate care and treatment.

## Data availability statement

The original contributions presented in the study are included in the article/supplementary material. Further inquiries can be directed to the corresponding author.

## Ethics statement

Ethical approval was not required for the study involving humans in accordance with the local legislation and institutional requirements. Written informed consent to participate in this study was not required from the participants or the participants’ legal guardians/next of kin in accordance with the national legislation and the institutional requirements.

## Author contributions

RP: Conceptualization, Investigation, Supervision, Writing – original draft, Writing – review & editing. EG: Conceptualization, Data curation, Project administration, Writing – original draft, Writing – review & editing. SB: Conceptualization, Writing – original draft, Writing – review & editing. SS: Conceptualization, Writing – original draft, Writing – review & editing. SA: Conceptualization, Writing – original draft, Writing – review & editing. KS: Conceptualization, Writing – original draft, Writing – review & editing. SR: Conceptualization, Writing – original draft, Writing – review & editing. KP: Conceptualization, Writing – original draft, Writing – review & editing. PJ: Conceptualization, Writing – original draft, Writing – review & editing. HM: Conceptualization, Writing – original draft, Writing – review & editing. GK: Conceptualization, Writing – original draft, Writing – review & editing. KA: Formal analysis, Methodology.

## References

[1] Society, A.C. Cancer facts & Figures 2023(2023). Available at: https://www.cancer.org/research/cancer-facts-statistics/all-cancer-facts-figures/2023-cancer-facts-figures.html.

[2] Society, A.C. Colorectal cancer facts & Figures 2020-2022(2020). Available at: https://www.cancer.org/content/dam/cancer-org/research/cancer-facts-and-statistics/colorectal-cancer-facts-and-figures/colorectal-cancer-facts-and-figures-2020-2022.pdf.

[3] Surveillance Epidemiology and End Results (SEER) Program. SEER cancer statistics review (CSR) 1975-2018(2021). Available at: https://seer.cancer.gov/csr/1975_2018/.

[4] Miller KD Nogueira L Devasia T Mariotto AB Yabroff KR Jemal A. Cancer treatment and survivorship statistics, 2022. CA: A Cancer J Clin. (2022) 72:409–36. doi: 10.3322/caac.21731 35736631

[5] Gabriel E Attwood K Al-Sukhni E Erwin D Boland P Nurkin S. Age-related rates of colorectal cancer and the factors associated with overall survival. J Gastrointest Oncol. (2018) 9:96–110. doi: 10.21037/jgo 29564176 PMC5848036

[6] Gabriel E Attwood K Al-Sukhni E Erwin D Boland P Nurkin S. Disparities in the age-related rates of colorectal cancer in the United States. Am Surg. (2017) 83:640–7. doi: 10.1177/000313481708300631 28637568

[7] Siegel RL Wagle NS Cercek A Smith RA Jemal A. Colorectal cancer statistics, 2023. CA Cancer J Clin. (2023) 73:233–54. doi: 10.3322/caac.21772 36856579

[8] Turab Mohammed RG Navpreet R Lemini R Wang K Agha A Neupane A. geographic and demographic disparities in colorectal cancer: A population-based study using national cancer database. Hematology/Oncology Stem Cell Ther. (2022) 16. doi: 10.56875/2589-0646.1061 37023221

[9] Zyczynski TM Shrestha R Cook NM. Analysis of racial disparities in time to treatment initiation and survival among patients with advanced cancers. J Clin Oncol. (2021) 39:127–7. doi: 10.1200/JCO.2020.39.28_suppl.127

[10] Nogueira LM Gupta S. Racial disparities in receipt of guideline-concordant care for early-onset colorectal cancer in the U.S. J Clin Oncol. (2022) 40:6544–4. doi: 10.1200/JCO.2022.40.16_suppl.6544

[11] Schermerhorn MC Grunvald MW O'Donoghue CM Rao RD Becerra AZ. Factors mediating racial/ethnic disparities in delayed treatment of breast cancer. Ann Surg Oncol. (2022) 29:7652–8. doi: 10.1245/s10434-022-12001-5 PMC9244454 35751007

[12] Tobin EC Dobbs E Deslich S Richmond BK. Race/ethnicity and social determinants of health and their impact on the timely receipt of appropriate operative treatment of colon cancer. Am Surg. (2024), 31348241241697. doi: 10.1177/00031348241241697 38551594

[13] Franssen RFW trous MTA Bongers BC Vogelaar FJ Janssen-Heijnen MLG. The association between treatment interval and survival in patients with colon or rectal cancer: A systematic review. World J Surg. (2021) 45:2924–37. doi: 10.1007/s00268-021-06188-z PMC8322003 34175967

[14] Mohammed T Gosain R Rana N Lemini R Wang K Agha A. Geographic and demographic disparities in colorectal cancer: A national cancer database analysis. Hematol Oncol Stem Cell Ther. (2023) 16:262–71. 10.56875/2589-0646.1061 37023221

[15] Gabriel E Thirunavukarasu P Attwood K Nurkin SJ. National disparities in minimally invasive surgery for pancreatic tumors. Surg Endosc. (2017) 31:398–409. doi: 10.1007/s00464-016-4987-6 27412124

[16] Bagaria SP Neville M Gray RJ Gabriel E Ashman JB Attia S. The volume-outcome relationship in retroperitoneal soft tissue sarcoma: evidence of improved short- and long-term outcomes at high-volume institutions. Sarcoma. (2018) 2018:3056562. doi: 10.1155/2018/3056562 30140165 PMC6081523

[17] Emmanuel Gabriel SN Attwood K Hochwald S Kukar M Nurkin S. Disparities in major surgery for esophagogastric cancer among hospitals by case volume. Am Surgeon. (2018) 9(3):503–16. doi: 10.21037/jgo.2018.01.18 PMC6006045 29998016

[18] Rana N Gosain R Lemini R Wang C Gabriel E Mohammed T. Socio-demographic disparities in gastric adenocarcinoma: A population-based study. Cancers (Basel). (2020) 12. doi: 10.3390/cancers12010157 PMC7016781 31936436

[19] Abbaszadeh Kasbi A Ashary MA Baksh M Nussbaum S Attwood K Gabriel E. Age-related survival outcomes for pancreatic cancer by age. Cancer Diagn Progn. (2022) 2:71–7. doi: 10.21873/cdp. PMC8962847 35400009

[20] Pita-Fernández S González-Sáez L López-Calviño B Seoane-Pillado T Rodríguez-Camacho E Pazos-Sierra A. Effect of diagnostic delay on survival in patients with colorectal cancer: a retrospective cohort study. BMC Cancer. (2016) 16:664. doi: 10.1186/s12885-016-2717-z 27549406 PMC4994409

[21] Robbins AS Siegel RL Jemal A. Racial disparities in stage-specific colorectal cancer mortality rates from 1985 to 2008. J Clin Oncol. (2012) 30:401–5. doi: 10.1200/JCO.2011.37.5527 22184373

[22] Miles A McClements PL Steele RJ Redeker C Sevdalis N Wardle J. Perceived diagnostic delay and cancer-related distress: a cross-sectional study of patients with colorectal cancer. Psycho-Oncology. (2017) 26:29–36. doi: 10.1002/pon.v26.1 26868950

[23] Seijo R Gomez H Freidenberg J. Language as a communication barrier in medical care for Hispanic patients. Hispanic J Behav Sci. (1991) 13:363–76. doi: 10.1177/07399863910134001

[24] Juckett G. Caring for Latino patients. Am Family Physician. (2013) 87:48–54. 23317025

[25] Simpson DR Martínez ME Gupta S Hattangadi-Gluth J Mell LK Heestand G. Racial disparity in consultation, treatment, and the impact on survival in metastatic colorectal cancer. J Natl Cancer Inst. (2013) 105:1814–20. doi: 10.1093/jnci/djt318 PMC4383284 24231453

[26] Siminoff L Thomson M Dumenci L. Factors associated with delayed patient appraisal of colorectal cancer symptoms. Psychooncology. (2014) 23:981–8. doi: 10.1002/pon.3506 PMC4604563 24615789

